# A Novel Potential Role for Monocytes Revealed by Single Cell Analysis of Immunotherapy Induced Immune Related Adverse Events

**DOI:** 10.3390/cancers14215407

**Published:** 2022-11-02

**Authors:** Zachary Garrison, Matthew Chang, Noah Hornick, Wesley Y. Yu, Jeffrey B. Cheng, Rajan P. Kulkarni

**Affiliations:** 1 Department of Dermatology, Oregon Health & Science University, Portland, OR 97239, USA; 2 Department of Dermatology, University of California San Francisco (UCSF), San Francisco, CA 94143, USA; 3 Department of Dermatology, Veterans Affairs Medical Center, San Francisco, CA 94121, USA; 4 Knight Cancer Institute, Oregon Health and Science University, Portland, OR 97239, USA; 5 Operative Care Division, U.S. Department of Veterans Affairs Portland Health Care System, Portland, OR 97239, USA; 6 Cancer Early Detection Advanced Research Center (CEDAR), Portland, OR 97239, USA

**Keywords:** cancer, immunotherapy, immune checkpoint blockade, autoimmunity, inflammation, immune related adverse event

## Abstract

**Simple Summary:**

Immunotherapy treatments have become one of the most popular options in cancer care. While their efficacy is well established, there is a significant risk of a variety of immune based side effects. These side effects, usually referred to as immune related adverse events, are well documented but poorly understood. To improve our understanding of the key cellular players involved in the development of these side effects, we have analyzed single cell sequencing data from PBMCs drawn from patients who developed skin immune related adverse events. Using that data, we have identified cellular population dynamic trends which point to potential mechanisms and pathways through which the side effects are occurring. This research is an important step in improving our understanding of the mechanisms that drive immune related adverse events.

**Abstract:**

Immune related adverse events (irAEs) are one of the leading causes of discontinuation of cancer immunotherapy treatment. Despite extensive research into the frequency and types of irAEs, little is known about the cell types and pathways through which these drugs cause the observed side effects. To identify cell types and pathways of interest, we have analyzed single cell sequencing data of PBMCs from patients who developed skin irAEs as a result of their immunotherapy treatment. Using Azimuth’s cell type identification software for PBMCs and GSEA pathway analysis, we found macrophage cell populations and reactive oxygen species related pathways to be upregulated. These results provide important groundwork to build a complete picture of the mechanisms which cause irAEs and finding ways to more effectively treat them.

## 1. Introduction

Within the last decade, checkpoint blockade immunotherapy (CBI) has quickly become one of the mainstays of cancer treatment. CBI-based therapies use antibodies to block immunosuppressive receptors, allowing for a “kickstart” to the host immune system. While these treatments have proven remarkably effective in their ability to promote immune responses targeting cancer, these benefits have come at the cost of variable disease responses and unintended side effects. Immune related adverse events (irAEs) are off target auto-immune responses induced by cancer immunotherapy. They are highly variable, affecting nearly any organ system across a wide range of severities. Most irAEs resemble known autoimmune or inflammatory disease states, and can develop weeks, months, or even years after the initiation of therapy. irAEs develop in roughly 40% of patients on PD-1 based CBI and 60–70% of patients on the CTLA-4 based treatment ipilimumab [1]. These adverse side effects are one of the leading causes for discontinuation of immunotherapy [2] and have garnered a significant amount of research attention. While irAEs are typically considered undesirable, with research focused on identifying markers to predict their development, some preliminary data has shown better long-term survival and/or disease response to therapy among patients who develop irAEs [2]. The skin is the most frequent site of irAE development; cutaneous irAEs affect as many as 71% of patients treated with CBI [3] and is the focus of our work here.

Given the inherent complexity surrounding the development of CBI-induced irAEs and the positive disease response they may signal, more research into the possible mechanisms driving irAEs is crucial. Through analysis of the immune cells responsible for these side effects, we aimed to better understand the mechanisms by which CBI produces its antitumor efficacy. Additionally, a better understanding of these mechanisms may improve our ability to treat CBI-induced irAEs without affecting the course of treatment. To begin parsing out these complex mechanisms, we have analyzed single cell RNA sequencing data of peripheral blood mononuclear cells (PBMCs) from patients profiled by Lozano et al. [4] which included both cancer patients on CBI who experienced skin irAEs as well as cancer patients on CBI who did not experience any irAE; both sets were compared to a healthy adult control population without a history of cancer [5]. These cells include 4734 cells from patients who developed skin irAEs while on immunotherapy, 5093 cells from melanoma patients on immunotherapy who did not develop any irAEs, as well as 18,531 cells from healthy adult patients. Of the cells from the irAE patients, 796 of the cells originate from patients with “mild” irAE conditions while 3938 originate from patients with “severe” irAEs. From these samples, we have identified key immune cell population dynamics which highlight the inflammatory mechanisms which may drive irAE development. Additionally, we have utilized the GSEA databank to identify pathway distinctions between the different irAE states. Collectively, this data shows the possible pathways through which irAEs may develop in the skin and potential targets to alleviate the most severe of these conditions.

## 2. Materials and Methods

We integrated two publicly available single-cell 5′ RNA-seq datasets from Gene Expression Omnibus (GEO), accession numbers GSE186144 (Lozano et al.) and GSE154386 (Waickman et al.). Only the data from pretreatment samples in the Waickman data were used in this study as a healthy control. Additionally, as the Lozano dataset was limited to the 2000 most variable genes, we subsetted the Waickman dataset to this feature set. In order to investigate skin irAEs specifically, the Lozano dataset was subsetted to only samples with no irAE and those that had exclusively skin irAE. The two datasets were then integrated using methods outlined in the Seurat v4 WNN integration vignette (including the SCTransform function for batch correction). Differential expression analysis was conducted using the FindAllMarkers function in Seurat v4.0.2 across IRAE severity levels as reported by Lozano et al. (0, 1, and 3) and an adjusted *p* value threshold of 0.05 (adjusted by Bonferroni correction). Genes returned by differential expression analysis were run through a Gene Set Enrichment Analysis (GSEA) alongside their log fold change values separated by irAE severity (clusterProfiler v3.18.1). The cells were then run through the cell type identification software Azimuth using their PBMC reference dataset to transfer cell labels (Figure S1). Differential cell type fractions were then tabulated and compared across irAE severity levels using R (Tables S1–S3).

## 3. Results

### 3.1. PBMC Population Dynamics

Single-cell RNA sequencing analysis of PBMCs from four different patients at baseline within the Lozano et al. dataset were used in this study. Of the four patients, there are two male patients who developed skin irAEs as a result of treatment with immunotherapy compared with a female and male patient who did not develop irAEs while receiving treatment. All patients within this cohort were diagnosed with metastatic melanoma and had varying responses to their treatments as outlined in Table 1 below. None of the patients included developed an irAE that was non-skin related nor were any receiving other cancer medications other than those listed.

### 3.2. Immune Cell Population Shifts

Variation between the healthy (non-cancer controls) and the melanoma patients who did not develop irAEs highlights notable increases in monocyte, Naïve B cell, and NK cell populations that may have occurred as a result of cancer development. Conversely, there are notable decreases to the CD4^+^ and CD8^+^ central and effector memory cell population respectively. These same trends are observed when comparing patients who went on to develop mild irAEs and those who developed no irAEs.

Monocytes have for some time been recognized as central modulators of both innate and adaptive immunity [6]. Terminology surrounding subtypes of myeloid antigen-presenting cell lineages can be controversial, but the groups identified by the workflow leveraged here are defined as CD14^+^ “classical” monocytes or as CD16^+^ “nonclassical” monocytes. Additional subsets are not included or distinguished by the classifier, but these cells are distinct from dendritic cells based on Azimuth’s interpretation of transcriptional phenotypes. Our analysis revealed slightly lower levels of CD16^+^ monocytes in the mild irAE population compared to the cancerous control population which had already shown a sharp increase from the healthy controls. Notably, the CD16^+^ monocyte population increased between the mild and severe irAE cells and represented a greater percentage of the total PBMC population compared to the cancerous control group. CD14^+^ monocytes also increased in population percentage in patients who went on to develop irAEs although that increase was greater in mild cases as compared to severe ones. This shift is notable given that the percentage of CD14^+^ cells appears to decrease between the non-irAE cancer patients and the mild case before rising again in the severe irAE population.

NK cell populations significantly increased in all cancer patients compared to the healthy control patients. This is notable given that the NK cells showed a slight negative trend with irAE severity although not large enough to negate the increase relative to the healthy controls. It is important to mention that the NK cells noted in Table 2 exclusively refer to CD56^dim^ as only CD56^dim^ NK cells were observed in all patient groups.

### 3.3. GSEA Pathway Analysis

**Figure 1:**
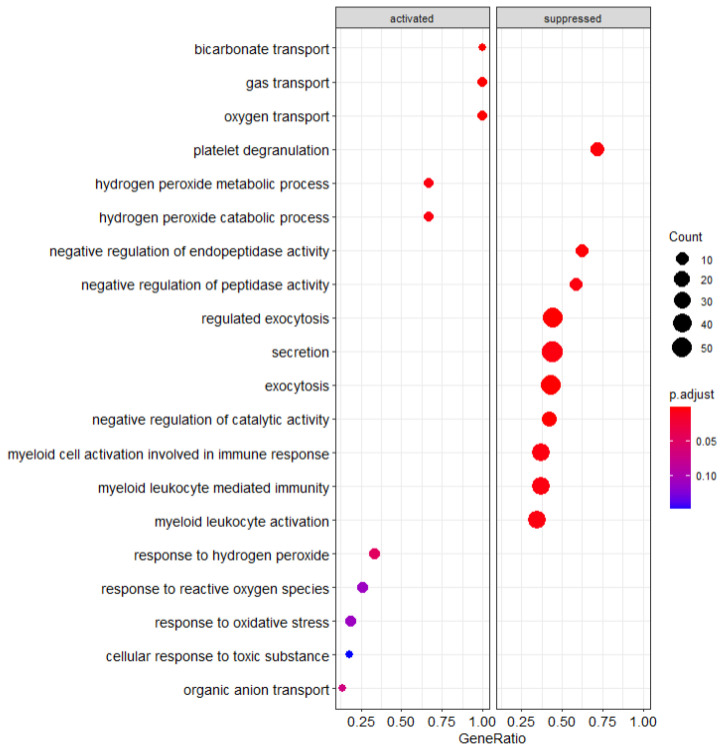
GSEA Analysis of healthy control patients compared to cancer patients.

By identifying gene expression changes associated with specific cellular pathways, we were able to identify mechanistic distinctions in the cellular functions of the PBMC populations we analyzed (Figure S2a–d). Through comparison of the patients who developed irAEs and the healthy control PBMCs, we have found specific pathways which are either upregulated or downregulated compared to the other PBMC populations. As seen from Figure 1 below, there are several statistically significant pathway changes observed within our GSEA output.

#### 3.3.1. Healthy Control PBMCs Compared to Cancer PBMCs

To separate the influence of cancer on the altered PBMC states, GSEA pathway analysis was performed comparing the healthy PBMC cohort to PBMCs originating from patients with cancer (both the irAE and non-irAE populations). Notable transport pathways are suppressed including exocytosis and secretion as shown in Figure 1. Suppression of myeloid activity is notable given its connection to the immune response and that suppression relative to the cancer cohort suggests increased activation in the cancer patient population.

#### 3.3.2. Mild vs. Severe irAE States

**Figure 2:**
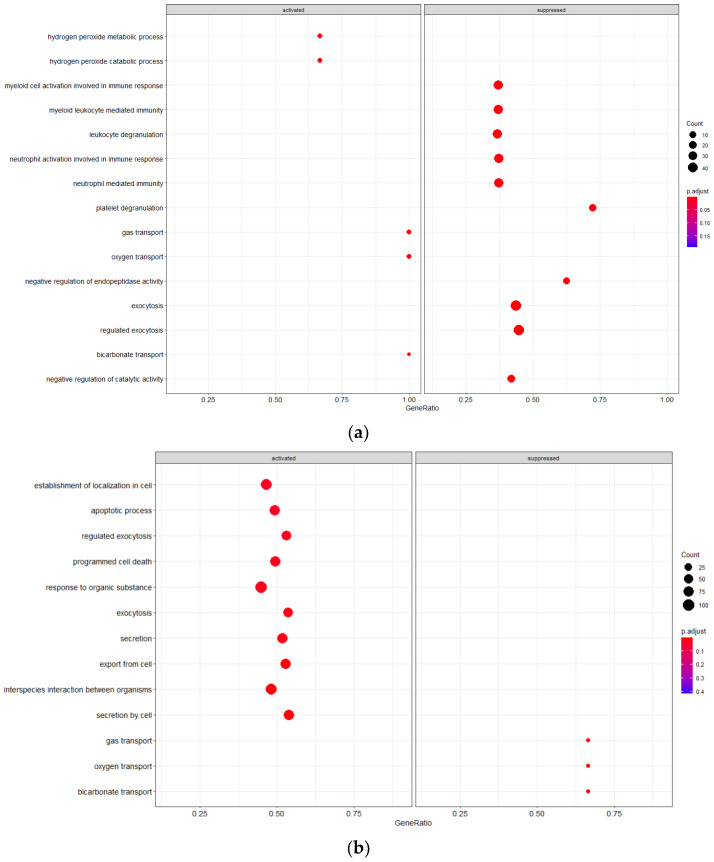
GSEA Analysis of Cellular Pathway Activation/Suppression (**a**) Mild irAE(**b**) Severe irAE.

Cell populations in the mild irAE group are distinct from the other conditions evaluated due to alterations in pathways suggestive of an innate immune response, including leukocyte degranulation, myeloid leukocyte mediated immunity, cell activation in immune response, and granulocyte activation (Figure 2). Gas transport pathways are also activated, a finding that is reversed in the severe irAE population. PBMCs from the mild irAE group show increased activation to hydrogen peroxide metabolism/catabolism suggesting an increased level of said chemical within the immune cell environment.

Severe irAE cells had additional pathway alterations of interest, including suppressed gas transport pathways and activation of cell death pathways such as programmed cell death and apoptotic process. Restriction of gas trafficking may point to the buildup of reactive oxygen species which would further potentiate active inflammatory responses. Conversely, the severe irAE cells demonstrated increased activation to secretion pathways (exocytosis, secretion, export from cell, secretion from cell).

## 4. Discussion

Analysis of these patient PBMCs provides valuable insight as well as the basis for additional new questions into the origins and mechanisms of immunotherapy-induced irAEs. Some of the observed trends, like those surrounding the CD4 and CD8 T cell populations, contradicted expected population shifts given prior studies describing inflammation in irAE [7,8]. This observation is notable given the preponderance of literature surrounding the role of effector memory cells in inflammatory states. CD8^+^ TEM cells have been seen to increase in irAE (although this observation has not yet been peer-reviewed), and tissue-resident memory cell transition to the effector state has been observed to drive CBI-related colitis [9]. There is precedent, however, to suggest that our observation of decreased circulating memory T cells may represent an alternative pathophysiologic mechanism. One study has identified a decrease in TEM cell populations among patients who developed irAEs although only a significant drop among CD4^+^ TEM cells was observed [10]. Another group recently found that circulating TEM (both CD4 and CD8) trended down in patients that developed irAE, while the opposite was true in patients whose tumors responded to their therapy [11]. Additional work is needed to determine whether these disparate patterns represent mechanistic differences between the irAE developing in different organ systems, between different patient contexts, or an alternative explanation is responsible.

One intriguing observation from the cell subsets is a substantial increase to NK cell populations in patients who developed irAEs. This observation is novel given the relative paucity of literature on NK cells in the context of immunotherapy. NK cells have been observed to act in both pro-inflammatory and immune-regulatory pathways. In a leukemia model, CD56^dim^ NK cells activated by inflammatory cytokines IL-12, IL-15, and IL-18 will produce elevated levels of IFN-γ as part of a memory-like response when subsequently exposed to cancer cells, suggesting a possible anticancer role for this population [12]. Other studies have observed increases in CD56^bright^ NK cells, which are thought to exert an immunoregulatory influence, in patients with autoimmune disease and chronic inflammation [13,14,15,16,17]. While research into CD56^dim^ NK cells is limited within the context of autoimmune responses, it has been implicated in the initiation of encephalomyelitis [18]. Additional work directed at these cell types specifically may yet reveal a significant role for them in the CBI-driven inflammation, either against tumor or against self.

Changes to cell population dynamics highlight an important role for monocytes in the observed inflammation. Both CD14^+^ and CD16^+^ monocytes have been ascribed a variety of roles in the context of malignancy, along both protumoral and antitumoral axes [19]. In the context of chronic inflammation, it has been suggested that CD14^+^ monocytes transition to the CD16^+^ phenotype, leading to a population shift in favor of the latter, and correlating with markers of tissue homing [20]. Direct inter-regulation between these populations has been proposed in patients with lupus [21]. In melanoma patients, it has been shown that untreated patients with untreated metastatic disease have decreased CD14^+^ monocytes in the peripheral blood compared to healthy controls, a finding recapitulated in our analysis, and that the phenotypes of these monocytes may be a predictor of disease response to CBI [22]. Although limited data exists thus far on monocyte dynamics in tissue during irAEs, early analyses of colitis patients point to an influx of monocytes with a phenotype resembling that of the CD14^+^ population reported here [23]. While additional studies are needed, the existing data clearly suggests an important role for these monocyte populations in irAE development.

Further evidence of their significance can be found within the pathway alterations demonstrated by our GSEA analysis. While many of the cell types implicated by these pathways (particularly granulocytes) are not included within the PBMC populations analyzed in this study, the observed pathway changes may be triggered by phagocytic functions within monocytes, or a more widespread response thorough which both lymphocyte and myeloid cell transcription is jointly regulated. Increased activation of secretion pathways points to the release of inflammatory factors/cytokines by these monocytes. The effects of these activities are likely further compounded by the increased response to pathways involving hydrogen peroxide, reactive oxygen species, and oxidative stress. While the specifics of how these mechanisms are initiated are not revealed from our data, they do identify several interesting components worthy of further investigation. Hydrogen peroxide is a member of a class of reactive oxygen species released during acute inflammation [24] that promotes secretion of IFN-γ [25]. In addition to increased levels of hydrogen peroxide, *PBMCs* from the mild irAE cohort also demonstrate activation of bicarbonate transport pathways, which are involved in instigation of inflammation through activation of JAK/STAT signaling to promote IFN- γ and macrophage proliferation [26]. Release of these inflammatory markers points to a potential mechanism through which these instigators may influence response to immune therapy observed in these patients. Shifts in hydrogen peroxide catabolism and gas transport pathways may be a reflection of the decreased myeloid activities described above, which have been extensively linked to hydrogen peroxide mediated inflammation [27]. Regardless, the significant upregulation of these pathways points to an important role of the aforementioned cellular components in irAE development which deserves further analysis. Single cell sequencing of a broader variety of cellular populations from patients with active irAEs in addition to protein analysis would reveal more about the specific inflammatory factors which are released as within the upregulated cellular populations. This would provide the most complete picture to date of the active mechanisms driving the CBI-induced autoimmune inflammation.

The analysis above also raises several questions about population shifts which seem to contradict expected trends in cancer as well as in other types of chronic inflammation. Prominent among these is the striking drop in CD8 TEM cells in irAE-prone patients relative to healthy controls. Most previous studies have observed increases in circulating CD8^+^ T cells relative to controls throughout the course of chronic inflammatory conditions like the ones within these patients. This feature of our findings, despite there being some precedent for it [6,11] is not fully understood and highlights the relative infancy of this area of cancer immunology research. With limitations in the feature availability in the irAE dataset, we are restricted with regard to the cell types we can address in this analysis. Future work in this area will require a more expansive analysis of both blood and tissue cell populations to capture a complete view of cell population changes as well as possible tissue uptake/release. Additionally, these studies must include comparisons of varying cancer types and immunotherapy regimens to enable controlling for other variables which may alter the irAE states considered in this study. We analyzed skin irAE patients exclusively in this study due to the high incidence of such side effects among patients on immunotherapy treatment. The literature detailed above highlights growing momentum in the expansion of our understanding of the off-target effects of immunotherapy; this work is one of the first of its kind single cell analysis as part of this movement.

Although many studies, including that of Lozano et.al., rely on PBMC, this blood fraction prevents analysis of many potentially significant cell types. Neutrophils are one such cell type, having well documented interactions with the PBMCs detailed above; they are noticeably absent from our analysis given its reliance on PBMC. Many of the aforementioned trends could implicate neutrophil involvement, including increased monocyte populations and increased cell secretion, phagocytosis, and degranulation pathways. Neutrophils have been extensively studied in relation to multiple disease states characterized by inflammation [28,29,30] and would be intriguing to analyze in a single cell format such as the one conducted here. This stresses the importance of further research which can provide a more complete picture of the immune cell populations and factors, including neutrophils, involved in irAE inflammation.

## 5. Conclusions

Our analysis identifies several important cell populations and accompanying pathways that show altered activity prior to the development of mild and/or severe cutaneous irAE. These findings propose potential pathways through which these side effects may develop. However, a significant portion of the pathogenesis of this process still remains to be elucidated. To better understand the overall pathway through which CBI initiates and potentiates both on- and off-target inflammation, it is crucial that studies expand their analysis to include a wide range of immune cell populations within the inflamed tissue itself. The CD14^+^ and CD16^+^ monocyte populations in addition to the CD4^+^ and CD8^+^ T memory cells discussed above are compelling targets for further analysis.

This study presents a framework for future single cell analysis through which we can begin to better understand the risk factors for CBI induced irAEs. Given that this study was focused exclusively on skin irAEs, further research will be necessary to distinguish the cellular differences in immunotherapy side effects within other tissues or associated with other tumor types.

## Figures and Tables

**Table 1:** Patient Demographics and Clinical Data.

Age (Years)	Sex	Metastatic Melanoma Subtype	ICI Regimen	Highest irAE Grade	Time to irAE or Follow-Up (Months)	Gastro-Intestinal (GI) irAE Status	ICI Response Status	Total Follow-Up Time (Months)
**72**	Male	Acral	Ipilimumab + Nivolumab	3	0.39	No	no durable benefit	9.36
**66**	Male	Sun-exposed	Ipilimumab + Nivolumab	1	14.85	No	durable clinical benefit	14.95
**80**	Female	Sun-exposed	Anti-PD1	0	18.37	No	not evaluable	18.37
**39**	Male	Indeterminate	Ipilimumab + Nivolumab	0	13.34	No	no durable benefit	13.34

PBMC populations were divided among three categories for comparison: healthy control (irAE 0), irAE low severity (irAE 1), and severe irAE (irAE 3). The irAE severity grades are assigned based on CTCAE v5.0 designations. The percentages of each cell type within its own group were compared across each irAE severity level as shown in Table 2 below:

**Table 2:** Cell Populations Percentages of Variable irAE Severity.

Cell Type	Healthy Control	irAE 0	irAE 1	irAE 3	Log Fold Change (Healthy–0)	Log Fold Change (0–1)	Log Fold Change (1–3)
Naïve B	2.865	11.60	7.915	6.196	2.02	−0.55	−0.35
CD14^+^ Monocyte	14.93	21.48	43.09	30.07	0.52	1.00	−0.52
CD16^+^ Monocyte	1.684	11.72	8.040	15.06	2.80	−0.54	0.91
CD4^+^ T Central Memory	35.32	26.59	21.98	26.54	−0.41	−0.27	0.27
CD8^+^ T Effector Memory	12.55	0.3927	0.1256	0.1270	−5.00	−1.64	0.02
Natural Killer Cell	3.057	23.72	18.22	14.02	2.96	−0.38	−0.38

Avg Predictive Score is a composite average of the mean predictive strength of the cell identification made for each cell type taken across all three cell populations.

## Data Availability

Data is available from GEO, accession GSE186144 and GSE154386.

## Supplementary Materials

The following supporting information can be downloaded at: https://www.mdpi.com/article/10.3390/cancers14215407/s1,: Figure S1. UMAPs of Cell Type Assignments; Figure S2. GSEA Analysis of Each Patient Group: (A) Healthy (B) Non-irAE Cancer Control (C) Mild irAE (D) Severe irAE. Table S1.Healthy Control Cell Assignments; Table S2. Mild irAE (irAE 1) Cell Assignments; Table S3. Severe irAE (irAE 3) Cell Assignments.
