# Supplementary material for: A Novel Potential Role for Monocytes Revealed by Single Cell Analysis of Immunotherapy Induced Immune Related Adverse Events
Source: Cancers (Basel). 2022 Nov 2;14(21):5407. doi: 10.3390/cancers14215407 (PMC9657437; doi:10.3390/cancers14215407)
Supplement: Supplementary file 1 [file cancers-14-05407-s001.zip › cancers-1920298-supplementary.pdf]

Figure S1. UMAPs of Cell Type Assignments

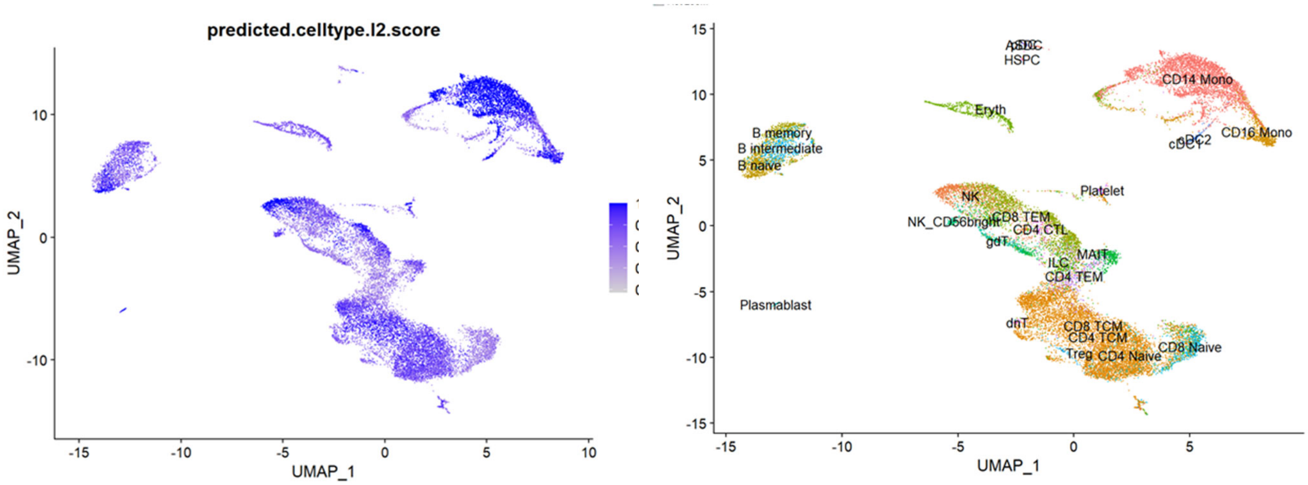

Figure S2. GSEA Analysis of Each Patient Group: (**a**) Healthy (**b**) Non-irAE Cancer Control (**c**) Mild irAE (**d**) Severe irAE

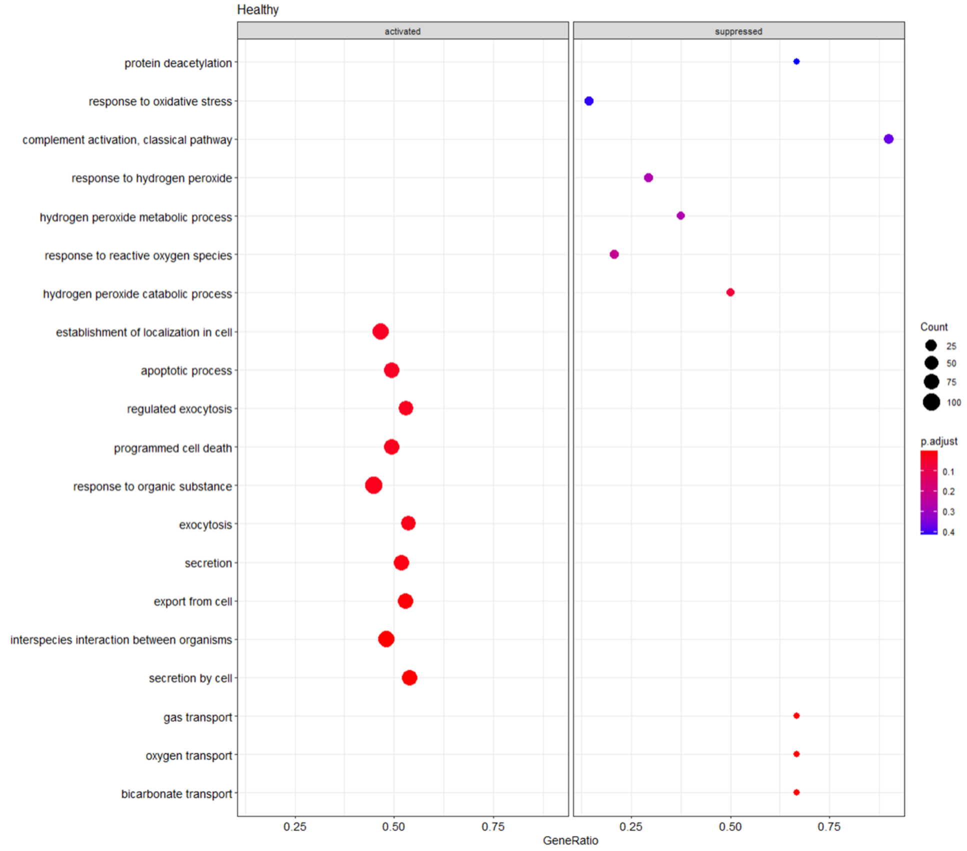

(a)

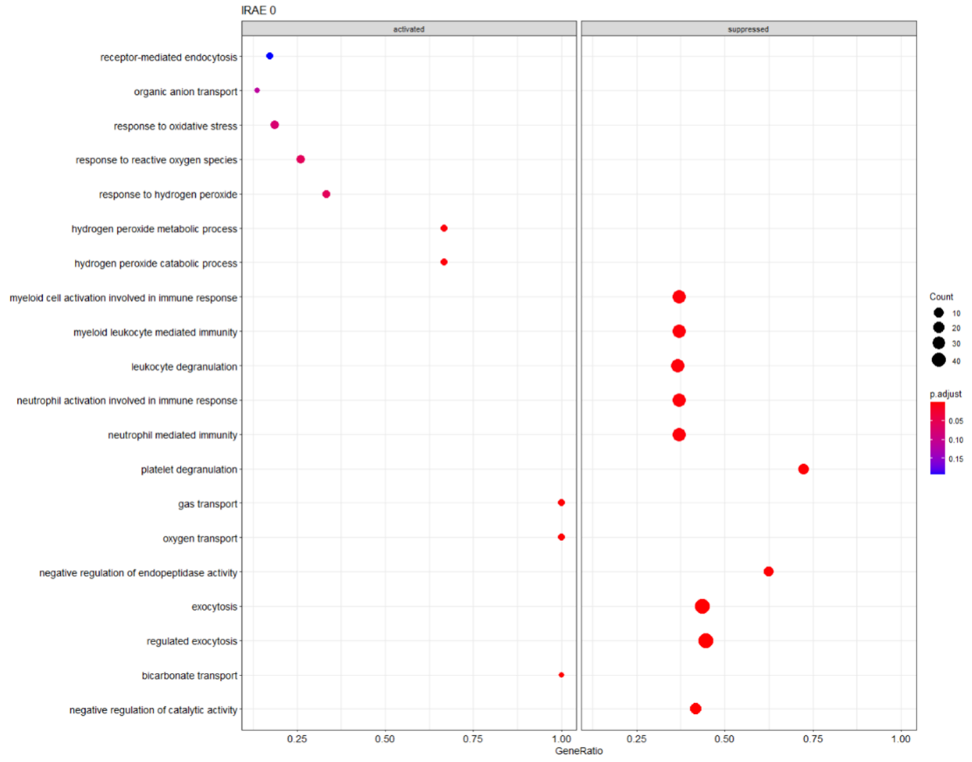
IRAE 0
activated
suppressed
receptor-mediated endocytosis
organic anion transport
response to oxidative stress
response to reactive oxygen species
response to hydrogen peroxide
hydrogen peroxide metabolic process
hydrogen peroxide catabolic process
myeloid cell activation involved in immune response
myeloid leukocyte mediated immunity
leukocyte degranulation
neutrophil activation involved in immune response
neutrophil mediated immunity
platelet degranulation
gas transport
oxygen transport
negative regulation of endopeptidase activity
exocytosis
regulated exocytosis
bicarbonate transport
negative regulation of catalytic activity
0.25
0.50
0.75
1.00
GeneRatio
Count
10
20
30
40
p.adjust
0.05
0.10
0.15

(b)

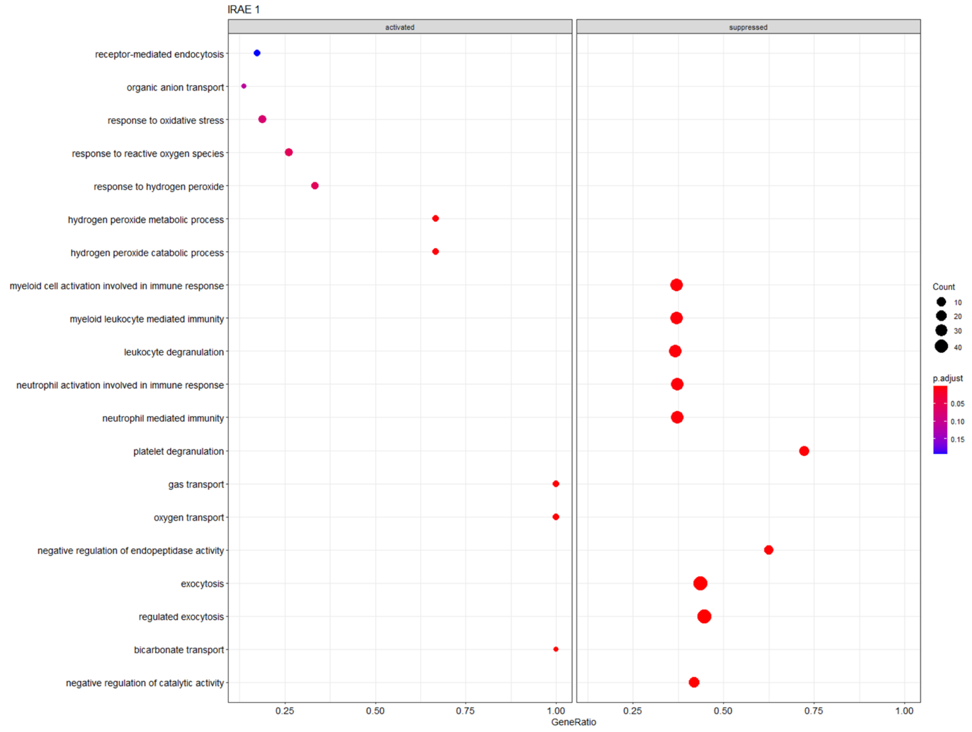
IRAE 1
activated
suppressed
receptor-mediated endocytosis
organic anion transport
response to oxidative stress
response to reactive oxygen species
response to hydrogen peroxide
hydrogen peroxide metabolic process
hydrogen peroxide catabolic process
myeloid cell activation involved in immune response
myeloid leukocyte mediated immunity
leukocyte degranulation
neutrophil activation involved in immune response
neutrophil mediated immunity
platelet degranulation
gas transport
oxygen transport
negative regulation of endopeptidase activity
exocytosis
regulated exocytosis
bicarbonate transport
negative regulation of catalytic activity
0.25
0.50
0.75
1.00
GeneRatio
Count
10
20
30
40
p.adjust
0.05
0.10
0.15

(c)

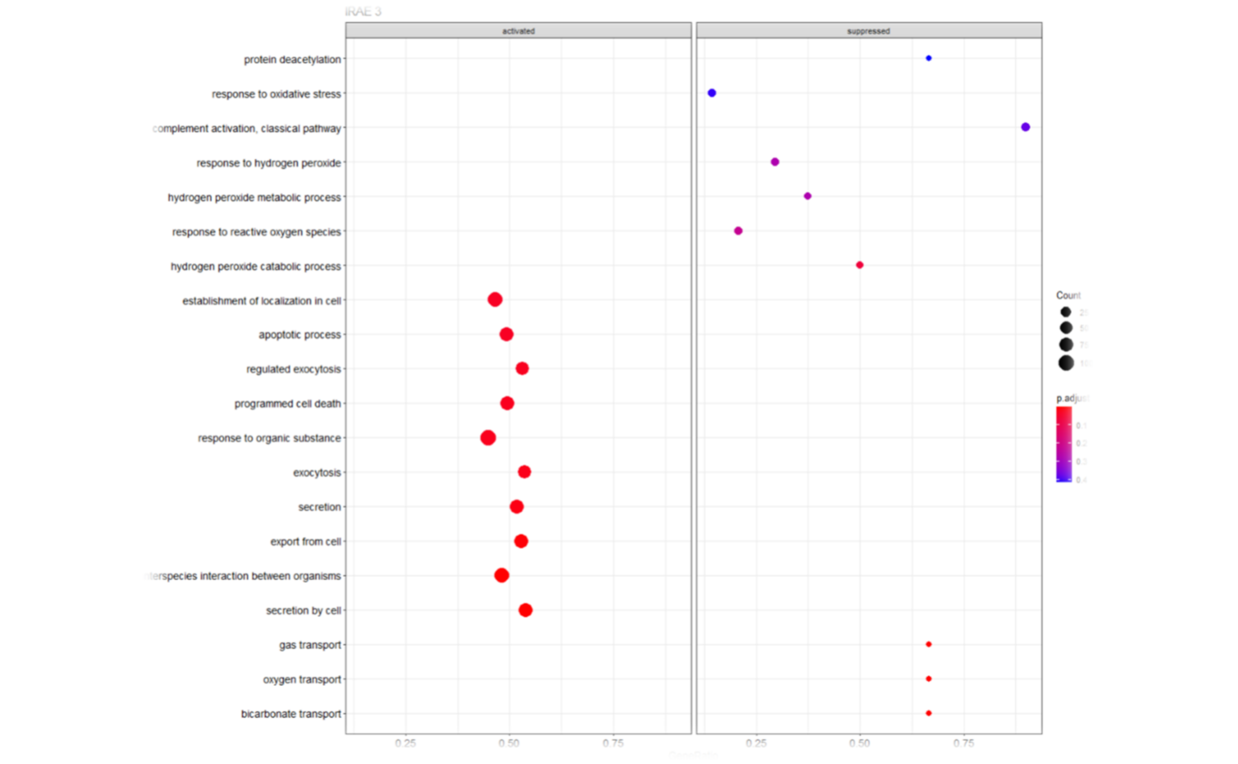

(d)

Table S1. Healthy Control Cell Assignments

Cell Type	Raw Cell Count	Percent	Mean_Predictive_Score
ASDC	7	0.03777454	0.49606021
B intermediate	533	2.8762614	0.72164779
B memory	569	3.07053046	0.71414872
B naive	588	3.17306136	0.77908905
CD14 Mono	2766	14.9263396	0.96251851
CD16 Mono	315	1.6998543	0.87853915
CD4 CTL	129	0.69613081	0.54173512
CD4 Naive	321	1.73223248	0.52838155
CD4 TCM	6535	35.2652312	0.77019956
CD4 TEM	521	2.81150505	0.53724872
CD8 Naive	584	3.15147591	0.5230922
CD8 TCM	355	1.91570881	0.56838908
CD8 TEM	2309	12.4602018	0.72269822
cDC1	10	0.05396363	0.64645274
cDC2	165	0.89039987	0.87735075
dnT	19	0.10253089	0.70318062
Eryth	699	3.77205763	0.76524918

gdT	291	1.57034159	0.58973207
HSPC	22	0.11871998	0.66089872
ILC	93	0.50186175	0.42069097
MAIT	721	3.89077762	0.64949903
NK	586	3.16226863	0.86661283
NK_CD56bright	154	0.83103988	0.7550185
pDC	74	0.39933085	0.56525878
Plasmablast	40	0.21585451	0.97932725
Platelet	42	0.22664724	0.90913164
Treg	83	0.44789812	0.57538285

Table S2. Mild irAE (irAE 1) Cell Assignments

Cell Type	Raw Cell Count	Percent	Mean_Predictive_Score
B memory	1	0.12562814	0.3423679
B naive	63	7.91457286	0.92317166
CD14 Mono	336	42.2110553	0.9465334
CD16 Mono	71	8.91959799	0.78433948
CD4 TCM	177	22.2361809	0.91976539
CD8 TEM	1	0.12562814	0.24030813
NK	147	18.4673367	0.98854359

Table S3. Severe irAE (irAE 3) Cell Assignments

Cell Type	Raw Cell Count	Percent	Mean_Predictive_Score
B naive	244	6.1960386	0.94493006
CD14 Mono	1184	30.0660234	0.89641667
CD16 Mono	593	15.0584053	0.78480405
CD4 TCM	1045	26.5363128	0.8135096
CD8 TCM	54	1.37125444	0.54774087
CD8 TEM	5	0.126968	0.41323044
Eryth	198	5.02793296	0.52524132
gdT	2	0.0507872	0.38763155
ILC	17	0.43169121	0.38664154
MAIT	41	1.04113763	0.31388433
NK	552	14.0172676	0.90407733
NK_CD56bright	3	0.0761808	0.32842437
